# After radiotherapy, do bone metastases from gastrointestinal cancers show response rates similar to those of bone metastases from other primary cancers?

**DOI:** 10.3747/co.v15i5.232

**Published:** 2008-10

**Authors:** A. Hird, E. Chow, D. Yip, M. Ross, S. Hadi, C. Flynn, E. Sinclair, Y.J. Ko

**Affiliations:** * Rapid Response Radiotherapy Program, Radiation Oncology, Odette Cancer Centre, University of Toronto, Toronto, ON; † Medical Oncology, Odette Cancer Centre, University of Toronto, Toronto, ON

**Keywords:** Bone metastases, gastrointestinal cancers, palliative radiotherapy, response

## Abstract

**Purpose:**

Reports investigating whether the response rates to palliative radiation therapy (rt) for painful bone metastases from gastrointestinal (gi) cancers are similar to rates for bone metastases from other primary cancer sites have been limited. The present study evaluated response rates for symptomatic bone metastases from gi cancers after palliative outpatient rt in the Rapid Response Radiotherapy Program (rrrp).

**Patients and Methods:**

We identified 69 patients with bone metastases from gi primaries who received palliative rt in the rrrp clinic during 1999–2006. We extracted records for 31 of these patients during 1999–2003 from an rrrp database that used the Edmonton Symptom Assessment Scale (esas). Record for the remaining 38 patients during 2003–2006 were extracted from an rrrp database that used the Brief Pain Inventory (bpi). Eligibility criteria for encryption in the two rrrp databases and for collection of patient demographic information (age, sex, primary cancer site, and Karnofsky performance status) were identical.

Response rates for this cohort of metastatic gi patients were then compared to rates for 479 patients receiving palliative rt for bone metastases from other primary cancer sites. Pain scores from the esas and bpi and data on analgesic consumption were collected at baseline and by telephone follow-up at 4, 8, and 12 weeks after rt for all patients. Complete (cr), partial (pr), and overall (cr+pr) responses were evaluated according to International Consensus Endpoints.

**Results:**

Assessment of the 69 patients with metastatic gi cancers revealed cr, pr, and cr+pr rates of 18%, 42%, and 61% at 4 weeks; 22%, 35%, and 57% at 8 weeks; and 50%, 21%, and 71% at 12 weeks for evaluable patients. The 479 evaluable patients with metastatic cancer from other primary cancer sites had cr, pr, and cr+pr rates of 25%, 27%, and 51% at 4 weeks; 26%, 22%, and 48% at 8 weeks; and 22%, 29%, and 51% at 12 weeks. No statistically significant differences were observed in rt response rates for bone metastases from gi cancers than from other primary cancer sites.

**Conclusions:**

After palliative rt, bone metastases from gi cancers demonstrate response rates that are similar to rates for metastases from other primary cancer sites. Patients with symptomatic bone metastases from gi malignancies should be referred for palliative rt as readily as patients with osseous metastases from other primary cancer sites.

## 1. INTRODUCTION

Bone metastases are a common complication of cancer. Bone is the third most common site of metastasis after lung and liver 1. Secondary bone tumours are particularly common in breast (47%–85%), prostate (54%–85%), lung (32%–40%), thyroid (28%–60%), and renal cell (33%–40%) cancers 1. Bone pain is the most common secondary symptom, with 50%–75% of individuals experiencing severe pain at some point during the course of their disease 2. As a result of severe pain, a patient’s quality of life, personal relationships, and psychological well-being can be significantly impaired. Palliative radiotherapy (rt) is an effective means of pain and symptom management for advanced cancer patients with bone metastases 3. Specifically, reports indicate that up to 90% of patients experience pain relief following palliative rt to symptomatic osseous metastases 3.

Randomized clinical trials have been conducted to determine the optimal dose fractionation for symptomatic relief of painful bony metastases. Multiple randomized controlled trials and three recent well-conducted meta-analyses concluded that pain relief is not significantly different with single-fraction or multiple-fraction palliative RT 4–6.

The development of skeletal metastases originating from gastrointestinal (gi) cancers is an uncommon event and typically presents in very advanced cases. The incidence of bone metastases in colorectal carcinomas ranges from 5.6% to 7.9% 7,8. Traditionally, the incidence of bone metastases from hepatocellular carcinoma (hcc) has been reported to be low (0%–5%) 9,10, but more recent investigations have reported an incidence of 6%–20% 11,12. Similarly, bone metastases from pancreatic carcinoma are becoming more common 13. Improvements in the diagnosis and treatment of these cancers have been credited for the observed increase in osseous metastasis 12,13. With this trend comes an immediate need for effective and timely symptomatic management within this cohort.

Most trials documenting rt response rates for bone metastases have accrued patients with breast, prostate, and lung primary cancers 4–6. Furthermore, bone metastases from breast and prostate cancers are typically considered highly radiosensitive 14 and therefore frequently responsive to a palliative rt intervention. Although no *a priori* reason exists to expect a different result for gi primary malignancies, the literature contains only limited reports concerning response rates for bone metastases from gi primaries after palliative rt 4–6. The present study examined response rates after palliative rt for the treatment of bone metastases from gi cancers to determine if response rates are significantly different from rates seen with rt of metastases from other primary cancer sites.

## 2. PATIENTS AND METHODS

The Rapid Response Radiotherapy Program (rrrp) at the Odette Cancer Centre developed a database for outpatients referred for palliative rt for bone metastases. The rrrp is an outpatient clinic that has been providing timely access to palliative rt since its start as a pilot program in 1996. Patients with advanced incurable cancer are referred to the rrrp for rapid access to palliative rt to relieve suffering and to improve quality of life 15. More than 60% of patients seen are referred for bone metastases in the rrrp^16^.

We identified 69 patients with bone metastases from gi primaries who received palliative rt in the rrrp clinic. We extracted records for 31 of these patients who were seen during 1999–2003 from an rrrp database that used the Edmonton Symptom Assessment Scale (esas). We extracted the remaining 38 patients who were seen during 2003–2006 from an rrrp database that used the Brief Pain Inventory (bpi). Eligibility criteria for encryption in both rrrp databases (the 1999–2003 version and the 2003–2006 version) were identical. Both study databases collected patient demographics [age, sex, primary cancer site, and Karnofsky performance status (kps)], site of rt, delivered rt dose, pain score (0–10), and oral morphine equivalent dose (omed). Merging the two sets of data for the study was therefore possible.

The research assistant approached all eligible patients seen in the rrrp. No preference was made regarding patients with a metastatic gi cancer diagnosis. We observed no statistically significant differences in age, sex, primary cancer site, or kps between the two gi cohorts (that is, the 31 patients accrued from 1999–2003 and the 38 patients accrued from 2003–2006; data not shown).

Ethics approval to conduct the analysis was obtained from the Sunnybrook Health Sciences Centre Research Ethics Board. Study participants provided written consent at the baseline assessment. Eligible patients had to be English-speaking and able to complete the symptom assessment, and had to have a previously demonstrated histologically or cytologically proven primary cancer site. All malignant histologies or cytologies were eligible. The presence of bone metastases corresponding to clinically painful areas had to have been confirmed by nuclear bone scan, computed tomography, or magnetic resonance imaging.

Patients were excluded from the study if a current or impending fracture was observed at the radiated site, or if a spinal cord compression (cc) existed at the time of enrolment into the study. Because radiologic evidence of bone metastases was required for eligibility for the trial, pathologic fracture, impending fracture, and spinal cc were identified according to the corresponding radiology report. Patients considered at “high risk for fracture” were not included in the investigation. Patients undergoing re-treatment were also ineligible for the study.

Using the esas and bpi questionnaires, participants were asked to rate their pain intensity on a categorical scale of 0 to 10 (0 indicating absence of pain; 10 indicating worst pain possible). Patient demographics and information on disease extent were collected at the baseline interview. Analgesic intake during the preceding 24 hours was recorded and converted into an omed. Because the bpi “worst pain” score has been shown to correlate most significantly with functional interference 17–23, we used this value to evaluate response to rt.

The research assistant conducted follow-ups at 1, 2, and 3 months (weeks 4, 8, and 12) post treatment. The follow-up interviews, conducted over the telephone or in person if the patient was scheduled for an appointment at the cancer centre, inquired about pain level and analgesic consumption.

Response rates were determined according to the International Consensus Endpoints 24 at 4, 8, and 12 weeks. “Complete response” (cr) was defined as a pain score of 0 at the treated site with no concomitant increase in analgesic intake (stable or reduced analgesics in daily omeds). “Partial response” (pr) was defined as

- a reduction in pain score of 2 or more points at the treated site on a scale of 0 to 10 without analgesic increase, or
- a reduction in analgesic use of 25% or more from baseline without an increase in pain.

Overall response (cr+pr) was calculated by adding the proportion of complete responders to the proportion of partial responders.

### 2.1 Statistical Methods

Descriptive statistics were recorded as percentages for proportions and as medians and ranges for parametric values. All statistical analyses were performed using the Statistical Analysis System (sas: SAS Institute, Cary, NC, U.S.A.). To evaluate rt response rates, we used a confidence interval of 95%, and we considered *p* values below 0.05 to be statistically significant.

## 3. RESULTS

For the period 1999–2003, we enrolled a total of 518 patients 25. Within this study population, we identified 31 patients whose bone metastases had gi cancer primaries. An additional 38 patients with bone metastases from gi cancers were extracted from the database spanning 2003–2006. Thus, we analyzed an overall sample of 69 gi metastatic patients. Table I shows the baseline patient demographics for this cohort, which contained 48 men (70%) and 21 women (30%). Median age of the cohort was 68 years (range: 37–89 years), and they had a median kps score of 60 (range: 40–90). The most common gi primary cancer sites were colorectal (68%), pancreatic (16%), and gastric (9%). The most prevalent dose fractionations were a single fraction of 8 Gy (49%) and 20 Gy in 5 fractions (44%). Table II lists the sites of radiation. Median baseline pain score at the irradiated site was 6 (range: 0–10). The mean and median omeds were 134 mg and 60 mg respectively (range: 0–1584 mg).

Removing the gi patients from the initial cohort of 518 patients produced a group of 479 patients with bone metastases from other primary cancer sites (Table III). This cohort was used for comparison of palliative rt response rates. It contained 253 men (53%) and 226 women (47%), whose median age was 69 years (range: 31–93 years). These patients presented with a median kps of 60 (range: 10–100). Most patients had primary lung (27%), breast (26%), or prostate (24%) cancer. Table IV lists the rt sites for this cohort. Median baseline pain score at the irradiated site was 6 (range: 0–10). The mean and median omeds were 101 mg and 30 mg respectively (range: 0–3600 mg). Patients were typically treated with a linear accelerator or a cobalt machine. Parallel-opposed fields were used to treat pelvis, limbs, and cranium; a direct oppositional field was used for the thoracic cage and spine. Follow-up assessments using the esas and bpi were conducted over the telephone or in person if the patient had been scheduled to return to the clinic for a follow-up with the clinician.

The numbers of gi patients who successfully completed follow-up assessments at 4, 8, and 12 weeks after rt were 33 (48%), 23 (33%), and 14 (28%) respectively. Table V shows response rates determined according to the International Consensus Endpoints 24. For evaluable patients, the cr, pr, and overall (cr+pr) rates were 18%, 42%, and 61% at 4 weeks; 22%, 35%, and 57% at 8 weeks; and 50%, 21%, and 71% at 12 weeks after rt.

For the cohort of 479 patients with bone metastases from other (that is, non-gi) primary cancer sites, the cr, pr, and cr+pr rates at 4 weeks after palliative rt were 25%, 27%, and 51% respectively. For evaluable patients at the 8-week follow-up, the rates were 26%, 22%, and 48%; and at 12 weeks, they were 22%, 29%, and 51%.

Using the Fisher exact test, we performed a comparison of the response rates for the two cohorts. Given a 95% confidence interval, we observed no statistically significant difference in terms of cr, pr, and cr+pr (Table V). These results suggest that bone metastases from gi cancers respond to palliative rt as well as do metastases from other primary cancer sites.

## 4. DISCUSSION

Many randomized trials and three recent meta-analyses of dose-fractionation rt for the palliation of painful bone metastases have concluded that single and multiple fractionations are equivalent^4^–^6^. We therefore combined the various dose fractionation schedules to assess the response rates of the entire cohort.

The response rate to rt is a function of endpoint. The International Consensus Endpoints take into account both pain score and analgesic consumption^24^. This method of evaluation lowers the cr and pr rates as compared with the pain-only endpoints. Moreover, as compared with analyzing pain score alone, using this method to calculate overall response produces a more accurate reflection of the true efficacy of rt. We integrated the International Consensus Endpoints into our analysis to promote consistency and to facilitate comparisons across trials. As predicted, the response rates reported in the present analysis are lower than the rates traditionally reported.

Literature on the effectiveness of palliative rt for bone metastases from gi cancers is limited 11,26–34. Large randomized controlled trials have not addressed response rates for gi bone metastases specifically. To our knowledge, no study directed at detecting differences in rt response rates between bone metastases from gi malignancies and those from other primary cancer sites has been conducted. Discrepancies regarding the perceived radiosensitivity of gi metastases may discourage the use of rt. The lengthened prognosis of gi cancer patients, coupled with the increased incidence of bone metastases from selected gi cancers^11^–^13^, creates an immediate need to maximize pain palliation and symptom management for this population.

We identified seven studies evaluating response rates for rt in the treatment of bone metastases from HCC 28–34. Additionally, we found a single study that investigated rt response in patients with spinal metastases from 11 hcc; however, the objective of treatment in this latter study was an existing or impending spinal cc. Given that spinal cc was indicated as an ineligibility criterion in the current study and in the referenced randomized clinical trials^4^–^6^,^25^, we therefore omitted the results of the aforementioned study from our comparison.

Each study that evaluated the efficacy of palliative rt in the treatment of symptomatic bone metastases from hcc concluded that pain relief was equal to that previously reported in other more general trials^28^–^34^. Within the metastatic hcc trials, sample sizes ranged from 12 to 57 evaluable patients, who were treated with a range of 12.5 Gy to 65 Gy. Reported response rates ranged from 73% to 94%.

We identified a single study investigating the rt sensitivity of bone metastases from pancreatic cancer^28^. Harada *et al.* evaluated 13 pancreatic cancer patients with 18 total sites of osseous metastasis. Pain relief was achieved in 12 of 13 patients (92%) with rt dosages ranging from 20 Gy to 30 Gy.

Two publications identified rt as an effective therapeutic tool in the management of bone pain for skeletal metastases originating from a colorectal primary^26^,^27^. Both investigations were retrospective reviews that summarized all colorectal carcinoma cases seen in the respective clinics. For 1970–1995, Kanthan *et al.* identified 5352 cases of colorectal carcinoma, 355 of which were identified to have osseous metastases^27^. A high preponderance (83.1%) had skeletal metastases in combination with metastases to lung, liver, or brain. The management of such metastases was palliative in nature. The authors identified rt as the most effective treatment for pain and adverse symptom control.

Similarly, for the period 1970–1980, Bonnheim *et al.* evaluated 1406 patients with primary colorectal adenocarcinoma^28^. In their cohort, 66 patients had skeletal metastases, and 71% had bone metastases in combination with other distant metastases. Again, the most effective treatment for palliation of the bone metastases was rt.

Our results compare favourably with the trial results^25^–^34^ and suggest no difference in effectiveness of rt for the symptomatic relief of pain originating from metastatic gi cancers to bone. Nonetheless, care should be used in the interpretation. Although the gi cancers in our patient population originated mainly from colorectal, pancreatic, and gastric primaries, published data on rt response in these malignancies are limited. As outlined, most of the published data reflect response in hcc metastases, and only a small proportion of our study population fell into this category.

Furthermore, inconsistent demographic factors (that is, age, performance status, and geographic location) and treatment-related factors (that is, rt dose) limit the comparison possibilities between the gi trials and the present investigation^25^–^34^. The dosing schedules used in the trials varied considerably. Known dosing schedules in the hcc trials ranged from 12.5 Gy to 65 Gy^28^–^34^, and Harada and colleagues delivered a median dose of 30 Gy to the 13 enrolled metastatic pancreatic cancer patients^28^. Within our study, 8 Gy in 1 fraction or 20 Gy over 5 fractions were the schedules most often used.

In addition, the absence of universal definitions of rt response makes comparison between investigations difficult. This difficulty has been documented not only in the present investigation, but also in existing publications evaluating the efficacy of palliative RT 24,26. A concise set of evidence-based endpoints has to be established to facilitate a comprehensive evaluation of pain relief following palliative rt in future clinical trials.

Our study population consisted only of outpatients receiving palliative rt in the rrrp clinic who met the study inclusion criteria; our sample is therefore not representative of all metastatic cancer patients receiving rt for bone metastases. A responder bias related to effect is therefore possible: patients who were available and willing to complete the follow-up assessment may have been those who responded favourably to the rt. Furthermore, the high drop-out rate is a limitation in the current study. Approximately half of the gi patients did not complete the 4-week follow-up assessment. The proportion of incomplete assessments further increased at 8 and 12 weeks after rt. Such a significant decline in available follow-up data creates a lack of power within the study. For example, in our trial, the 71% response rate for gi patients at week 12 is reported as being not significantly different from the 51% response rate for patients with other primary cancer sites. Although a 20% difference in response is large, a limited sample size created limited power to detect statistical significance.

Nonetheless, our series is one of the largest reporting on radiosensitivity of bone metastases from gi malignancies. From our analysis, pain relief resulting from rt in this group of patients is no different from the relief achieved with rt for metastases from all other primary sites. Although our data demonstrate limited power because of the small sample size and high drop-out rate, we hope our investigation will spark further research in the palliative field regarding the radio-sensitivity of metastatic gi malignancies. Such research is becoming progressively more significant as the prognosis of gi patients improves, and the incidence of skeletal metastases from these cancers increases. In the meantime, we encourage practitioners to refer gi cancer patients with symptomatic bone metastases to palliative rt as readily as they do breast or prostate cancer patients with bone metastases.

## Figures and Tables

**TABLE I:** Characteristics of patients (*n* = 69) receiving palliative radiotherapy (rt) to symptomatic bone metastases originating from gastrointestinal cancers

Characteristic	Value
Sex [*n* (%)]	
Male	48 (70)
Female	21 (30)
Age at RT (years)	
Median	68
Range	37–89
Worst pain	
Median	6
Range	0–10
Oral morphine equivalent dose (mg)	
Mean	134±229
Median	60
Range	0–1584
Primary cancer site [*n* (%)]	
Colorectal	47 (68)
Pancreatic	11 (16)
Stomach	6 (9)
Esophageal	3 (4)
Liver	2 (3)
Karnofsky performance status at first visit	
Median	60
Range	40–90
Dose fractionation [*n* (%)]	
8 Gy in 1 fraction	34 (49)
20 Gy in 5 fractions	30 (44)
Others	5 (7)

**TABLE II:** Sites of radiation for gastrointestinal cancer patients (*n* = 69)

Radiation site	n (%)
Spine	22 (32)
Pelvis	18 (26)
Extremities	13 (19)
Rib cage	5 (7)
Other	11 (16)

**TABLE III:** Characteristics of patients (*n* = 479) receiving palliative radiotherapy (rt) to symptomatic bone metastases originating from other (that is, non-gastrointestinal) primary cancers

Characteristic	Value
Sex [*n* (%)]	
Male	253 (53)
Female	226 (47)
Age at RT (years)	
Median	69
Range	31–93
Worst pain	
Median	6
Range	0–10
Oral morphine equivalent dose (mg)	
Mean	101±239
Median	30
Range	0–3600
Primary cancer site [*n* (%)]	
Lung	131 (27)
Breast	127 (26)
Prostate	117 (24)
Unknown	33 (7)
Multiple myeloma	29 (6.)
Renal cell/kidney	17 (4)
Bladder	11 (2)
Other	14 (3)
Karnofsky performance status at first visit	
Median	60
Range	10–100
Dose fractionation [*n* (%)]	
8 Gy in 1 fraction	193 (40)
20 Gy in 5 fractions	199 (42)
Others	87 (18)

**TABLE IV:** Sites of radiation for non-gastrointestinal cancer patients (*n* = 486^a^)

Radiation site	n (%)
Spine	140 (29)
Pelvis	150 (31)
Extremities	100 (21)
Rib cage	27 (6)
Other	69 (14)

a The overall (that is, non-gastrointestinal) patient population consisted of 479 individuals. However, some patients received palliative radiation treatment to more than one site of bony metastasis. Therefore, 486 metastatic sites in total were irradiated.

**TABLE V:** Comparison, by Fisher exact test at weeks 4, 8, and 12, of evaluable and non-evaluable response rates^a^ in bone metastases patients, 479 with metastases from non-gastrointestinal primaries and 69 with metastases from gastrointestinal (gi) primaries

	Non-gi primary cancer (n=479)			gi primary cancer (n=69)			
	Patients (n)	Evaluable [% (95% CI)]	Non-evaluable [% (95% CI)]	Patients (n)	Evaluable [% (95% CI)]	Non-evaluable [% (95% CI)]	p Value
Week 4		[n=251 (52%)]			[n=33 (48%)]		
cr	62	25 (19–31)	13 (10–16)	6	18 (7–35)	9 (3–18)	0.52
pr	67	27 (21–33)	14 (11–17)	14	42 (25–61)	20 (12–32)	0.07
cr+pr	129	51 (45–58)	27 (23–31)	20	61 (42–77)	29 (19–41)	0.36
Week 8		[*n=*219 (46%)]			[*n=*23 (33%)]		
cr	57	26 (20–32)	12 (9–15)	5	22 (7–44)	7 (2–16)	0.80
pr	48	22 (17–28)	10 (7–13)	8	35 (16–57)	12 (5–22)	0.19
cr+pr	105	48 (41–55)	22 (18–26)	13	57 (34–77)	19 (10–30)	0.51
Week 12		[*n=*188 (75%)]			[*n=*14 (20%)]		
cr	42	22 (17–29)	9 (6–12)	7	50 (23–77)	10 (4–20)	0.05
pr	54	29 (22–36)	11 (9–14)	3	21 (5–51)	4 (1–12)	0.76
cr+pr	96	51 (44–58)	20 (17–24)	10	71 (42–92)	15 (7–25)	0.17

a Response rates were calculated according to the proportion of evaluable (that is, the number of patients contacted at the respective follow-up assessment) to non-evaluable (that is, total number of) patients in the cohort.

ci = confidence interval; cr = complete response; pr = partial response; cr+pr = overall response.
